# COVID-19 pneumonia: a pictorial review of CT findings and differential diagnosis

**DOI:** 10.1186/s43055-021-00415-2

**Published:** 2021-01-25

**Authors:** Fattane Shirani, Azin Shayganfar, Somayeh Hajiahmadi

**Affiliations:** Department of Radiology, School of Medicine, Isfahan University of Medical Sciences, Isfahan, Iran

**Keywords:** COVID-19 pneumonia, CT findings, Differential diagnosis

## Abstract

**Background:**

The gold standard for verifying COVID-19 mostly depends on microbiological tests like real-time polymerase chain reaction (RT-PCR). However, the availability of RT-PCR kits can be known as a problem and false negative results may be encountered. Although CT scan is not a screening tool for the diagnosis of COVID-19 pneumonia, given the widespread acquisition of it in the pandemic state, familiarity with different CT findings and possible differential diagnosis is essential in this regard.

**Main text:**

In this review, we introduced the typical and atypical CT features of COVID-19 pneumonia, and discussed the main differential diagnosis of COVID-19 pneumonia.

**Conclusions:**

The imaging findings in this viral pneumonia showed a broad spectrum, and there are no pathognomonic imaging findings for COVID-19 pneumonia. Although CT scan is not a diagnostic and screening tool, familiarity with different imaging findings and their differential diagnosis can be helpful in a rapid and accurate decision-making.

## Background

In December 2019, a series of pneumonia cases emerged in Wuhan, China. Thereafter, the disease rapidly spread worldwide. Accordingly, based on WHO declaration, it became pandemic on March 11, 2020. The gold-standard test for COVID-19 diagnosis is real-time reverse transcription–polymerase-chain-reaction (RRT-PCR). Although imaging is not considered as a diagnostic tool for COVID-19 and most radiology professional organizations and societies unanimously disagree with performing screening computed tomography (CT) for the identification of COVID-19, a large number of chest computed tomography (CT) scans have been done worldwide to assess the severity and extension of the lower respiratory tract involvement [1]. Therefore, radiologists should be more familiar with CT scan findings of COVID-19 pneumonia and its differential diagnosis. Typical imaging features are those frequently seen, which are more specific for COVID-19 pneumonia on the basis of the literature review in the current pandemic [2]. These findings include peripheral bilateral ground glass opacities (GGO) with or without consolidation or crazy-paving pattern, reverse halo sign or other findings related to organizing pneumonia (OP), and multifocal GGO of the rounded morphology with or without consolidation or visible intralobular lines (crazy-paving). However, this viral pneumonia showed imaging findings which are less specific for COVID-19 pneumonia or uncommonly reported; these findings can be classified as indeterminate or atypical findings. Correspondingly, they include diffuse GGO with no clear distribution, isolated lobar or segmental consolidation with no GGO, discrete small nodules (centrilobular, “tree in-bud”), lung cavitation, and smooth interlobular septal thickening with pleural effusion [3].

The Dutch radiological society developed a categorical CT scheme-CORADS-to assess the suspicion for pulmonary involvement of COVID-19 based on features seen at unenhanced chest CT. It ranges from CO-RADS category 1 with very low level of suspicion to CO-RADS category 5 with very high level of suspicion. If none of five categories can be assigned secondary to incomplete or insufficient images, its considered as CO-RADS category0. CO-RADS category 6 indicates proven COVID-19 by positive RT-PCR test results (Table 1) [4].

**Table 1:** Overview of CO-RADS categories

CO-RADS category	Level of suspicion for pulmonary involvement of COVID-19	CT findings
0	Uninterpretable	Technically insufficient CT scan
1	Very low	Normal or non-infectious
2	Low	Consistent with other infection rather than COVID-19
3	Indeterminate	Unclear whether COVID-19 is present
4	High	Suspicious for COVID-19
5	Very high	Typical for COVID-19
6	Proven case	Positive RT-PCR for COVID-19

**Fig. 1:**
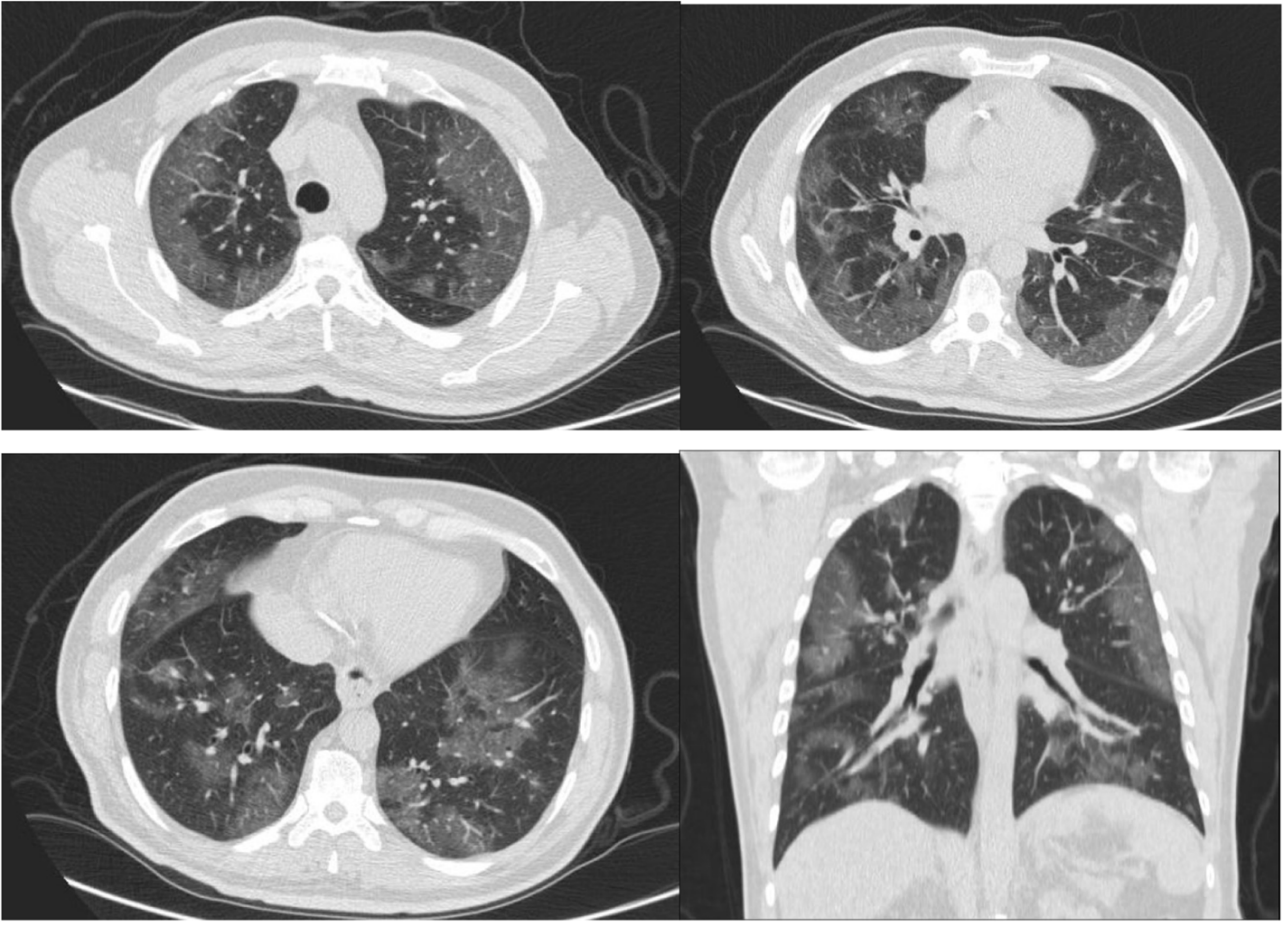
COVID-19 pneumonia: thin section CT shows bilateral multifocal subpleural and peribronchial GGO, semiqutitative score:18

Moreover, there is a semi-quantitative scoring system for quantitatively estimating parenchymal abnormality on the basis of the area involved. Each one of the five lung lobes was individually evaluated and weighted based on parenchymal involvement scored on a scale ranged from 0 to 5, with 0 indicating no involvement, 1 indicating less than 5% involvement, 2 indicating 5–25% involvement, 3 indicating 26–49% involvement, 4 indicating 50–75% involvement, and 5 indicating more than 75% involvement. The total CT score was the sum of the individuals’ lobar scores, which was ranged from 0 (no involvement) to 25 (the maximum involvement) (Fig. 1) [5]. In this review, we introduced different CT features of COVID-19 pneumonia and discussed the main differential diagnosis. Recognition of these features could help radiologists to have a rapid and accurate diagnosis.

Henceforth, each one of the imaging feature of COVID-19 pneumonia with RT-PCR is described, as confirmed at our referral hospital for COVID-19 pneumonia. Images illustrated in differential diagnosis were extracted from the images’ archive of the Department of Radiology.

## Main text

### Typical findings

#### Peripheral ground-glass opacities

On HRCT, GGO refers to the area of the increased lung opacity in which underlying bronchovascular markings are not obscured [6].

**Fig. 2:**
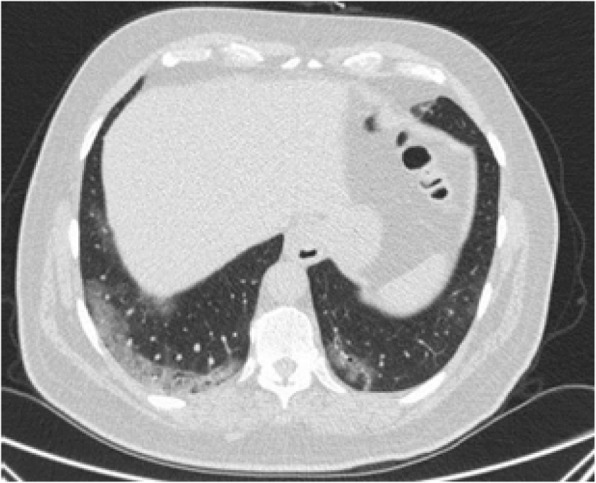
COVID-19 pneumonia: thin section CT shows bilateral subpleural GGO and septal thickening

GGO is the most common manifestation (40–83%) of COVID-19 pneumonia. Right and left lower lobes are most commonly involved. Multilobar subpleural GGO is seen in most cases. However, COVID-19 pneumonia may manifest as unilateral GGO even before the onset of symptoms with rapid evolution into diffuse, bilateral disease [7] (Fig. 2).

**Fig. 3:**
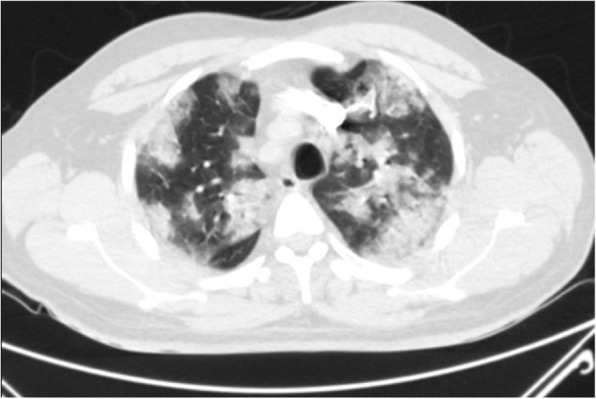
Diffuse alveolar damage: thin section CT shows patchy areas of GGO and consolidation in the lung periphery in upper lobes

**Fig. 4:**
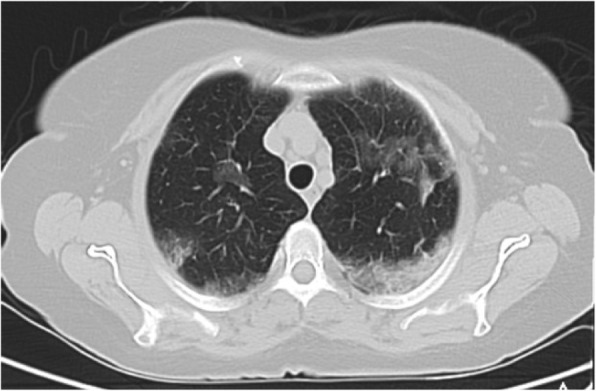
Simple pulmonary eosinophilia: thin section CT shows consolidation and GGO involving mainly the peripheral regions of both upper lobes

**Fig. 5:**
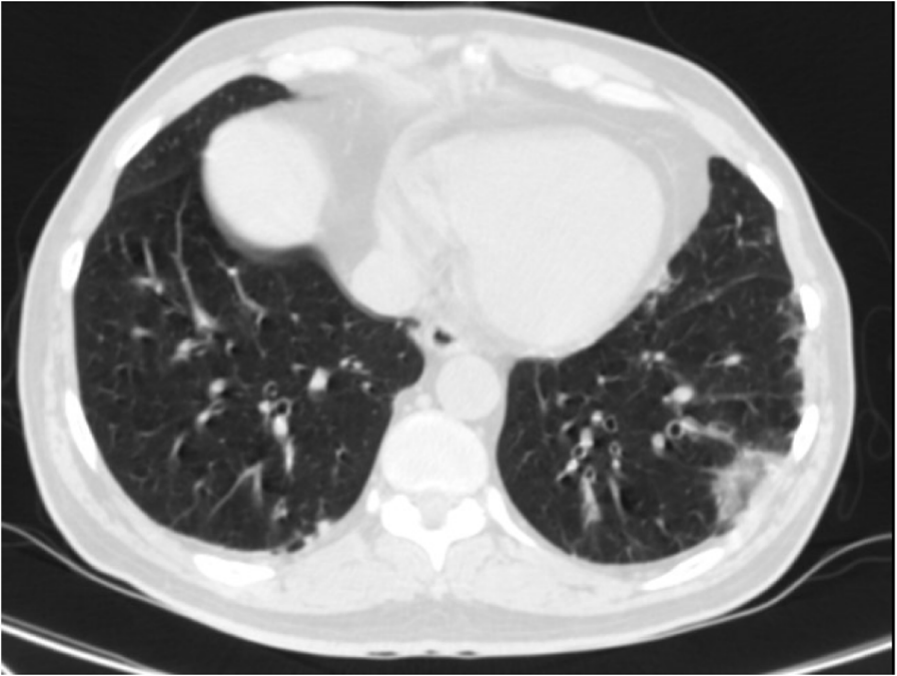
Influenza A viral infection: thin section CT shows subpleural GGO in left lower lobe

Differential diagnosis of GGO in the thin section CT was shown to be correlated with the clinical setting. In an acute setting, clinical history is more important than the distribution of GGO; however, in a chronic setting, its distribution is helpful in narrowing down the differential diagnosis. Notably, in patients with acute symptoms, some entities with peripheral distribution such as diffuse alveolar damage [6] (Fig. 3), simple pulmonary eosinophilia (Loffler syndrome) [8] (Fig. 4), and some viral pneumonia like influenza A have been described [9] (Fig. 5).

**Fig. 6:**
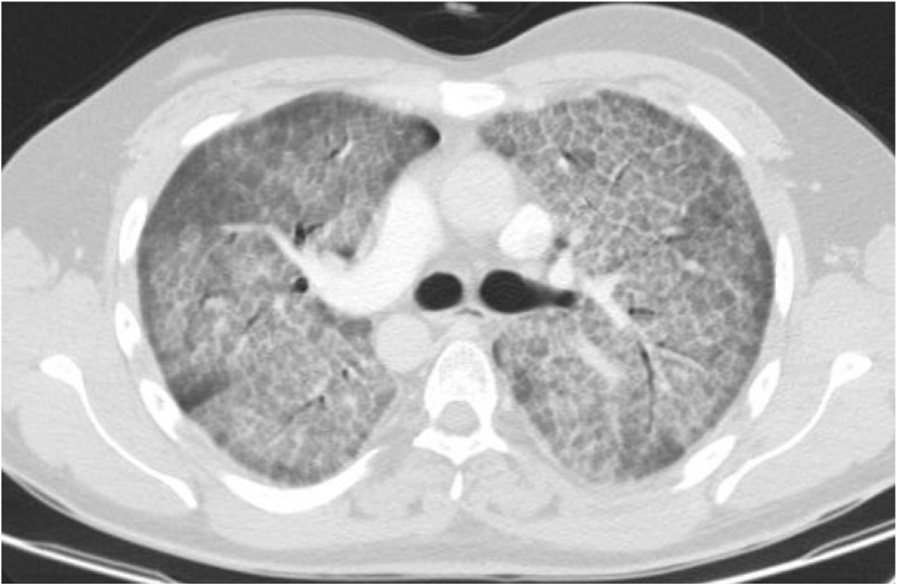
Pulmonary alveolar proteinosis: thin section CT shows bilateral GGO and reticulation (crazy-paving appearance)

In some patients with ground-glass opacity on HRCT, superimposition of a reticular pattern resulted in crazy paving appearance. This pattern was initially recognized in patients with pulmonary alveolar proteinosis (PAP) (Fig. 6), and it may also be seen in other differential diagnoses of GGO [10].

**Fig. 7:**
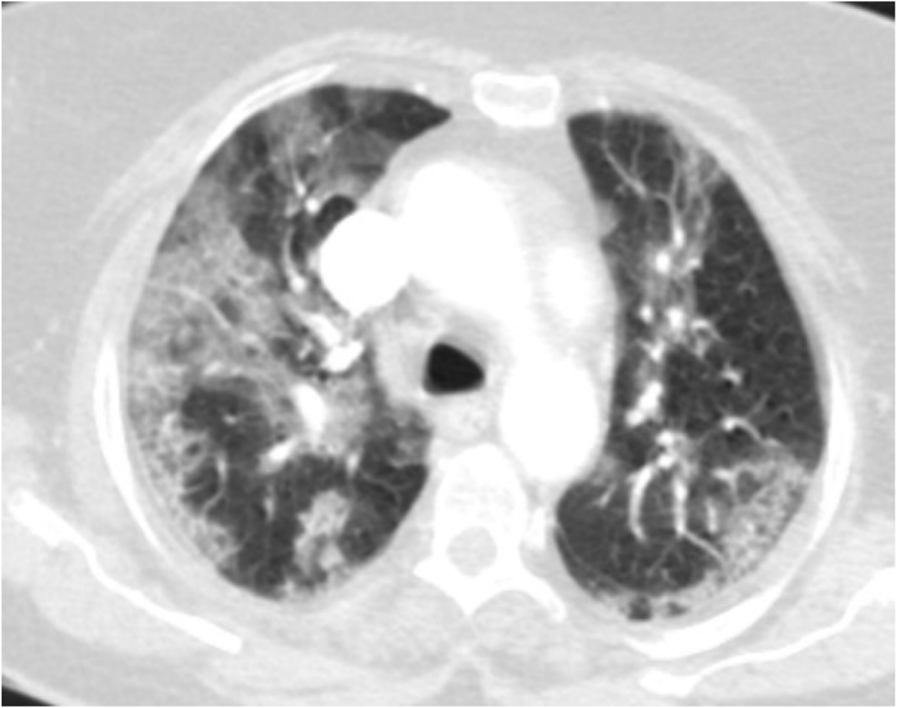
COVID-19 pneumonia: thin section CT shows bilateral multifocal subpleural and peribronchial GGO and reticulation (crazy -paving appearance)

Some recent studies have also reported the crazy paving pattern in 5–36% of patients with COVID-19 pneumonia [11]. This appearance can be considered as an indicator of disease progress or it may be recognized as secondary to the peak stage of COVID-19 pneumonia (Fig. 7) [5].

#### Reverse halo appearance

Reverse halo sign, also known as the Atoll sign, can be defined as a round or ovoid GGO surrounded by the complete or crescent ring of consolidation [6].

**Fig. 8:**
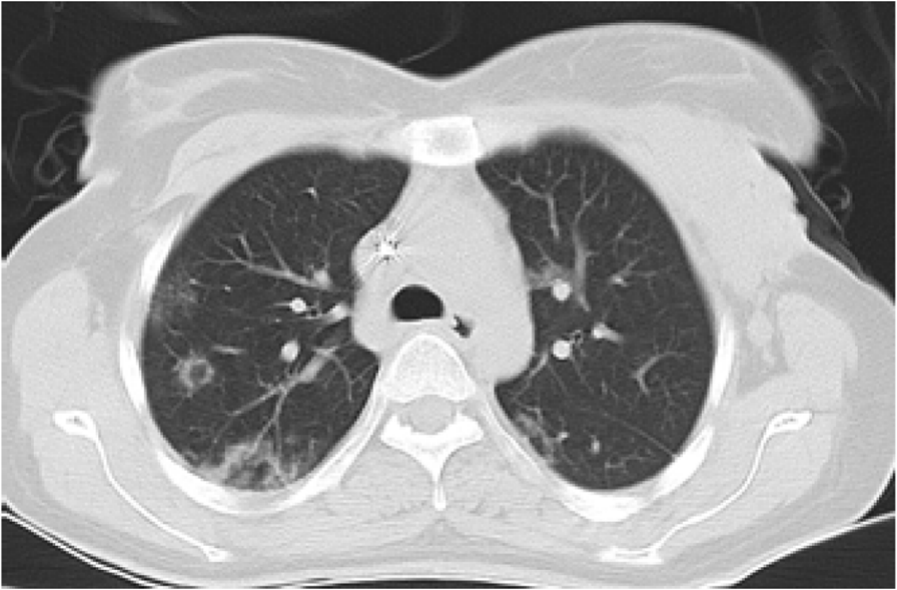
COVID-19 pneumonia: thin section CT shows multifocal peripheral GGO, with reverse halo appearance

This sign has been reported in several COVID-19 cases (Fig. 8). Moreover, it is assumed to be secondary to disease progression, which can consequently result in the development of consolidation around GGO or lesion absorption with the consequent decreased central density [12].

**Fig. 9:**
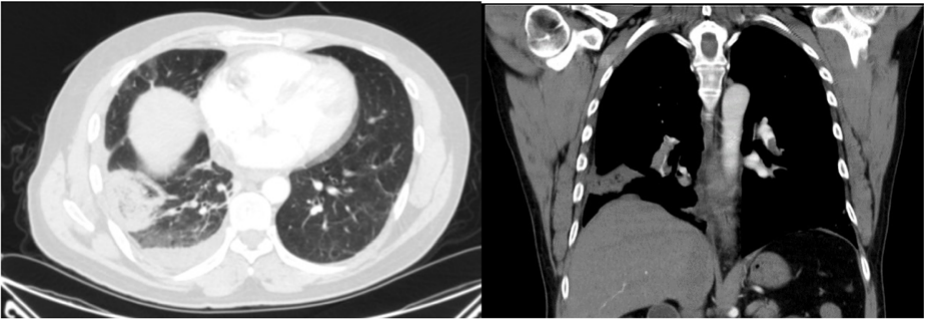
Pulmonary embolism: thin section CT in axial plane in lung window shows subpleural consolidation with reverse halo sign(RHS), coronal image in mediastinal window show filling defect in lower lobes pulmonary arteries suggestive for pulmonary embolism

Initially, the presence of reverse halo sign was believed to be specific for OP, but its differential diagnosis has broadened, such that we can remember it with mnemonic VISCERAL: Vasculitis, Infection, Sarcoidosis, Cryptogenic organizing pneumonia, Emboli, Radiation, and radioablation, Adenocarcinoma and Lymphomatoid granulomatosis. From non-infective processes, one important differential diagnosis that must be kept in mind is pulmonary infarction; in the patients with appropriate clinical history and laboratory data, in the presence of reverse halo sign on the non-contrast CT scan, the prompt evaluation of pulmonary vasculature, in contrast-enhanced CT with pulmonary thromboembolism(PTE) protocol, is essential (Fig. 9) [13, 14].

**Fig. 10:**
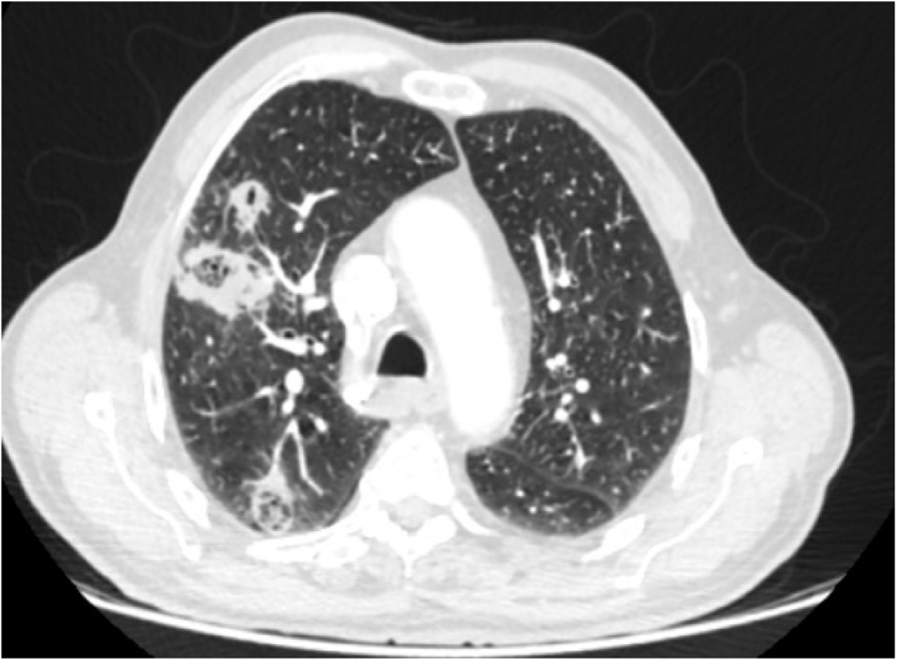
Pulmonary zygomycosis: thin section CT shows multiple nodules with reversed halo sign (RHS) in the right upper and lower lobes

In the infective process, this appearance is not specific. In immunocompromised patients, when there is a suspected fungal infection, the reverse halo sign is more frequently expected in mucormycosis than in invasive pulmonary Aspergillus [15] (Fig. 10). Additionally, in active tuberculosis, the Atoll sign can be expected, but it shows a nodular appearance [16].

#### Findings of organizing pneumonia

**Fig. 11:**
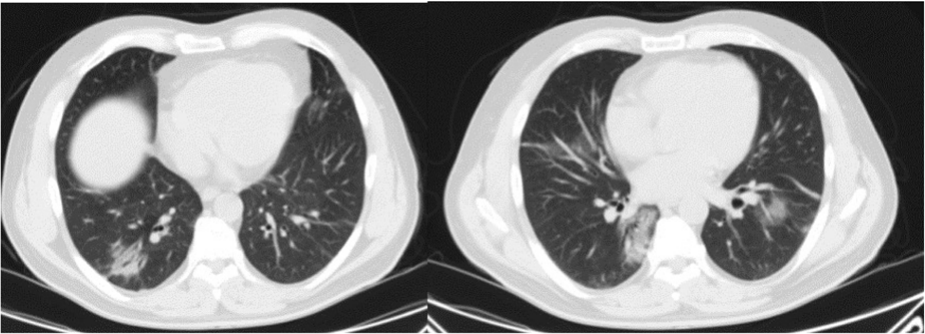
COVID-19 pneumonia: thin section CT shows mass-like peribronchovascular and subpleural consolidation in lower lobes

OP is an inflammatory non-infectious abnormality, which can be idiopathic (cryptogenic OP) or secondary to the connective tissue disease, drug toxicity, infection, toxic inhalation, immunologic disorders, and graft versus host disease (GVHD). The most typical findings of the high-resolution computed tomography (HRCT) of OP include nodular or mass-like consolidation with peribronchovascular and subpleural predominance. The findings show more severity in the lower lobes [6]. Based on an expert panel review published in MARCH 2020, the most common reported CT findings in COVID-19 pneumonia are secondary to lung injury with organizing pneumonia pattern [17, 18] (Fig. 11). One finding that is highly suggestive of OP is the Atoll sign or reversed halo sign as described earlier in the previous paragraph [6].

### Indeterminate-atypical findings

#### Diffuse GGO without clear distribution

**Fig. 12:**
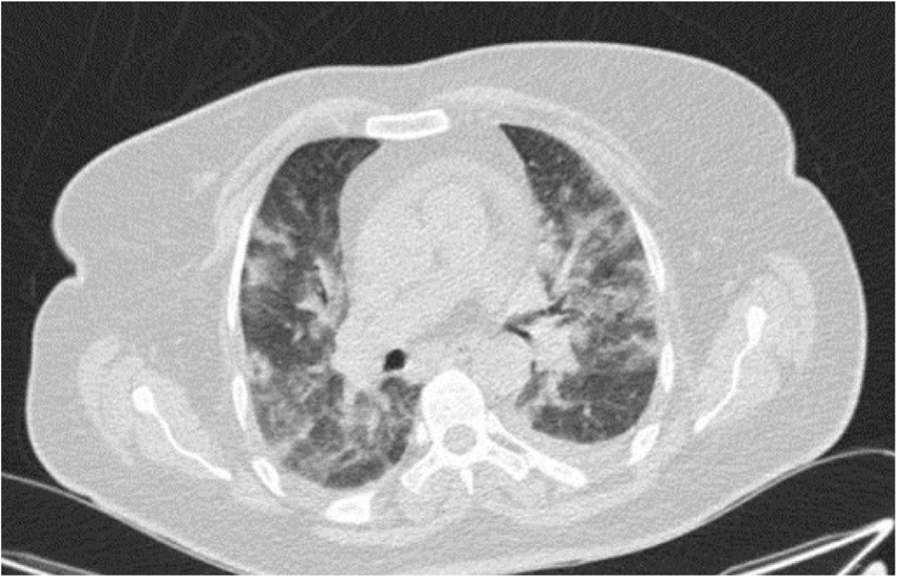
COVID-19pneumonia: parahilar GGO without round configuration and bilateral pleural effusion, despite indeterminate findings for COVID-19 pneumonia, RT-PCR test was positive for COVID-19

**Fig. 13:**
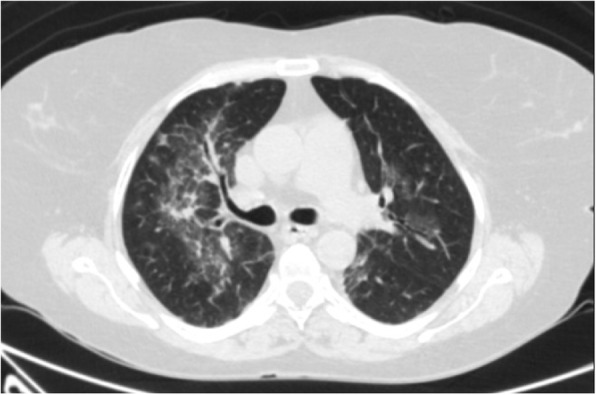
Pneumonia due to P jiroveci infection: thin section CT shows parahilar GGO with reticulation (crazy-paving appearance)

**Fig. 14:**
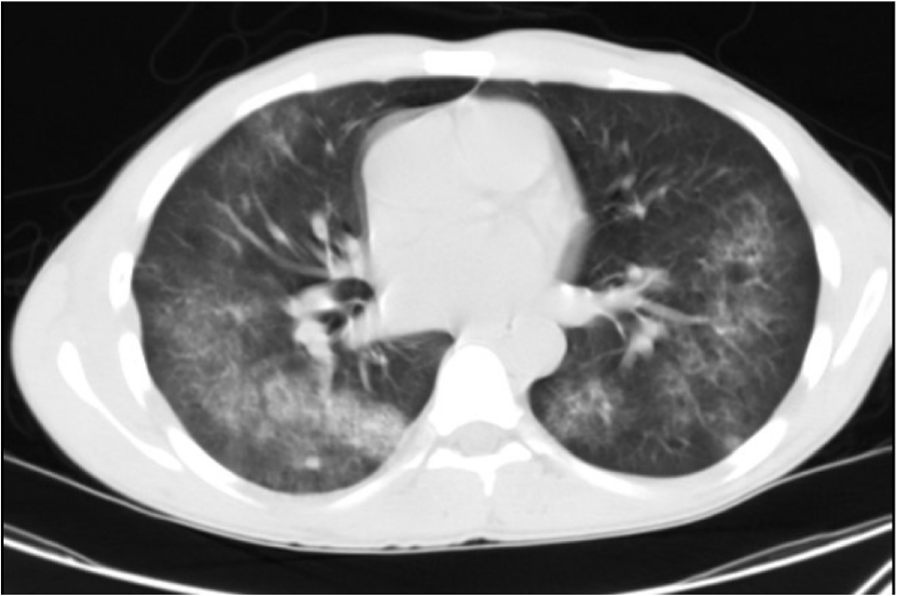
Pulmonary hemorrhage: thin section CT shows diffuse bilateral parahilar GGO without round configuration

This is a common finding in COVID-19 pneumonia (Fig. 12); however, it has been encountered in various diseases such as pneumocystis infection (Fig. 13), and diffuse alveolar hemorrhage (Fig. 14). So, differentiating these entities by imaging alone is difficult in such circumstances [3].

#### Nodular opacities with ground-glass halo

**Fig. 15:**
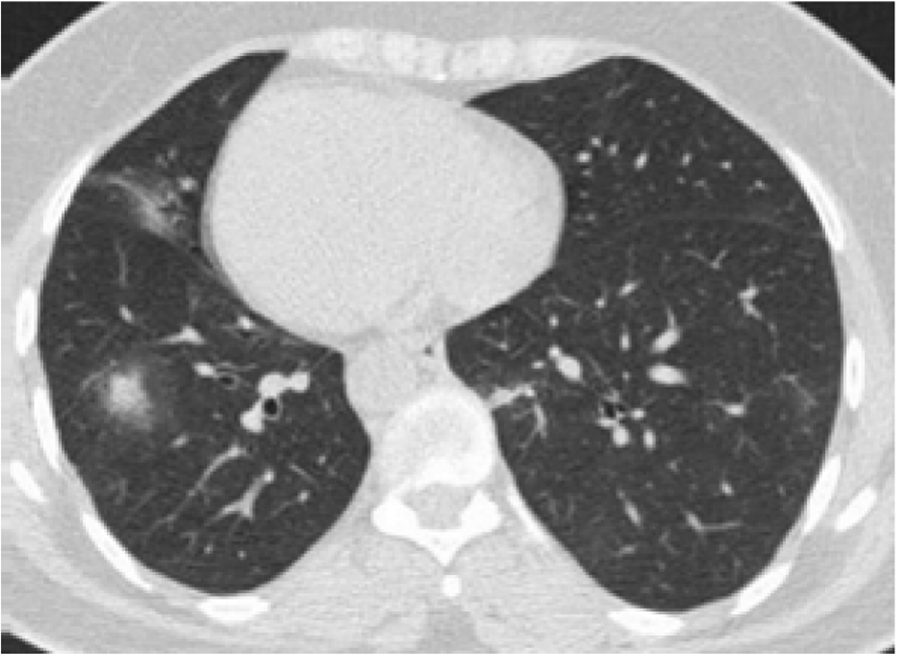
COVID-19 pneumonia: thin section CT shows nodular opacity with ground glass halo

Halo sign is defined as a condition in which GGO surrounds the central nodule or mass. This finding, in the thin section CT, is pathologically attributed to the presence of hemorrhage [19]. Although this appearance is unusual in COVID 19 pneumonia, it has been reported in some cases [20, 21] (Fig. 15). However, the main pathological stimulus of this manifestation still remains unknown.

**Fig. 16:**
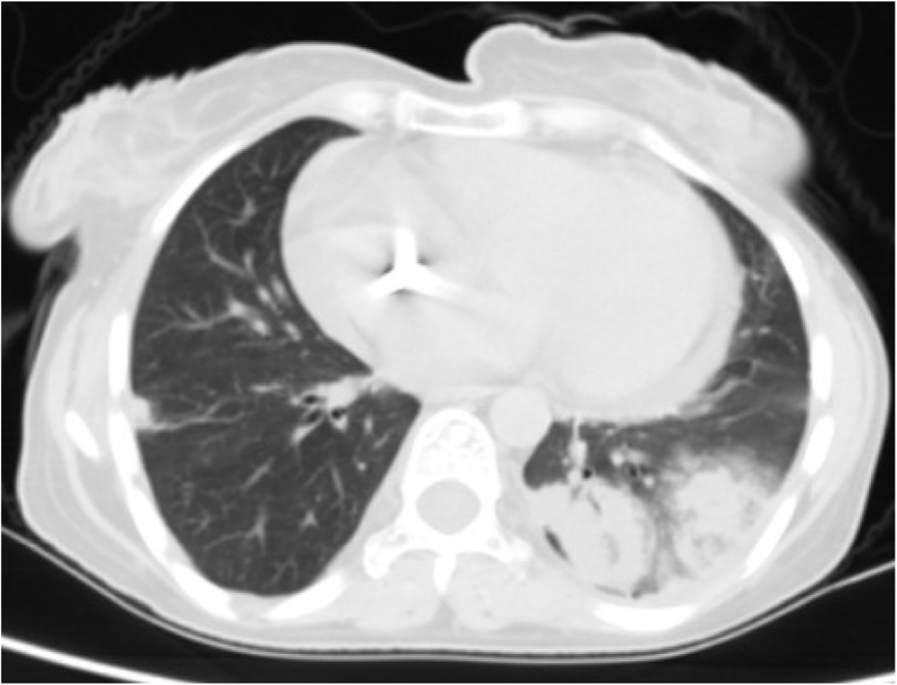
Angioinvasive aspergillosis represented by halo: thin section CT shows nodular area of consolidation with surrounding ground-glass opacities (halo sign) in left lower lobe

Differential diagnosis is broad, which includes infectious and noninfectious entities. Many infectious diseases including septic emboli, tuberculosis, herpes simplex virus, varicella-zoster virus, influenza, and invasive pulmonary Aspergillus (Fig. 16) have been described in this regard [19].

#### Focal consolidation

On HRCT, area of the increased lung opacity with obscuration of underlying bronchovascular markings refers to consolidation [6].

**Fig. 17:**
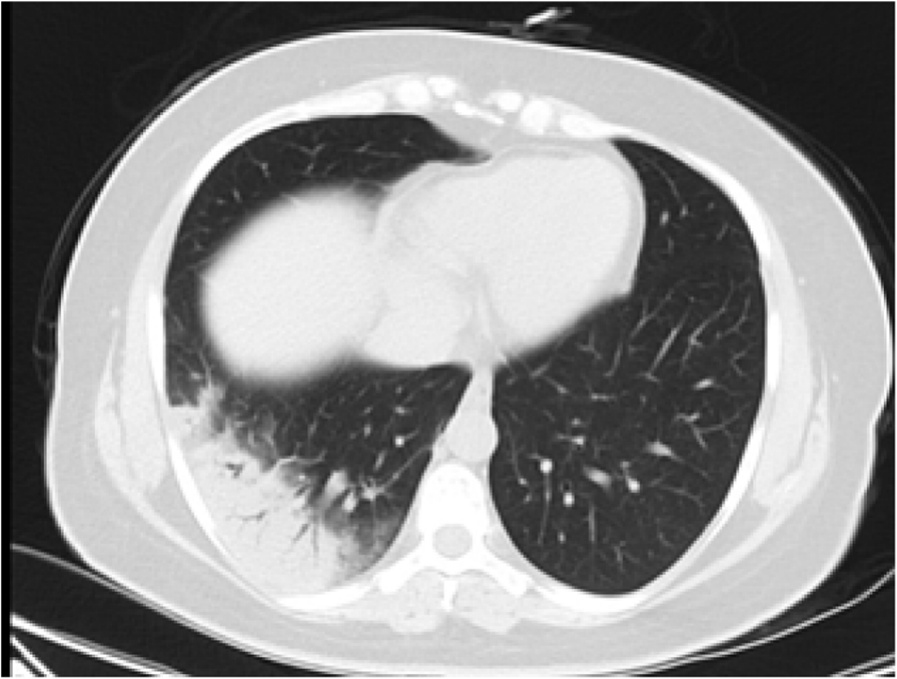
COVID-19 pneumonia: thin section CT shows non segmental parenchymal consolidation with airbronchogram in right lower lobe.

Parenchymal consolidation with multifocal, patchy, or segmental distribution in subpleural and peribronchovascular regions has been reported in 2–64% of cases infected with this disease [12]. In COVID-19 pneumonia, when there is a longer time interval between the symptom onset and CT scan, or in those patients older than 50 years old, lesions usually show a more consolidative appearance [22]. In COVID-19 pneumonia, unilateral lesions can be observed, especially immediately after the onset of symptoms, in asymptomatic patients or in those with minimal symptoms. Accordingly, they were described in 18.7% of cases in a meta-analysis of 34 studies performed on 4121 patients [23]. In these situations, sublobar pneumonia could be simulated (Fig. 17).

**Fig. 18:**
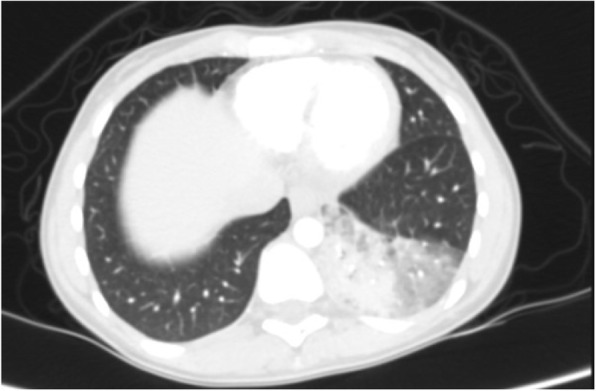
Streptococcus pneumonia: thin section CT shows segmental consolidation and GGO in left lower lobe

Differential diagnosis of parenchymal consolidation is related to the patient history; in an acute clinical setting, the infective process is highly considered. Notably, most bacterial pneumonias such as Streptococcus (Fig. 18) and Klebsiella pneumonia appear as lobar consolidation [24].

#### Centrilobular nodules

**Fig. 19:**
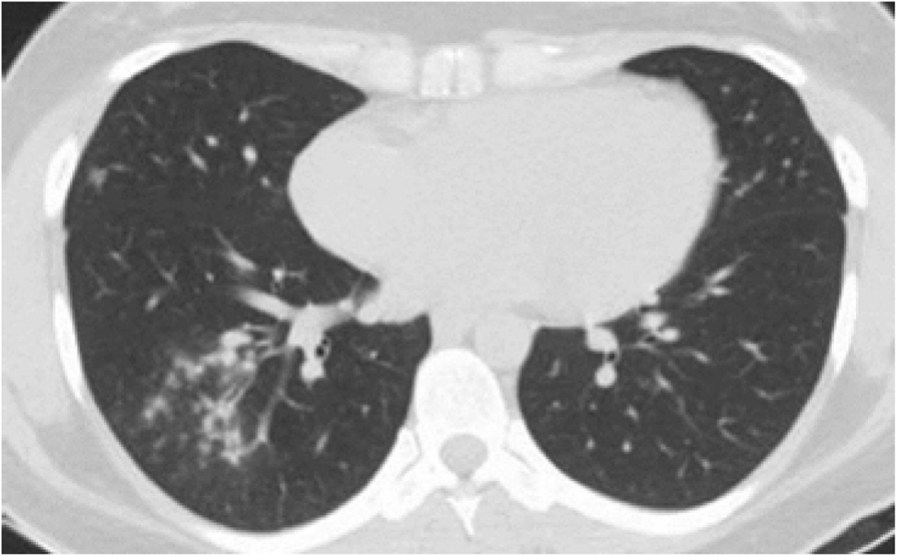
COVID-19 pneumonia: thin section CT shows peribronchial thickening and centrilobular nodules with tree in bud appearance. despite atypical findings for COVID-19 pneumonia, RT-PCR test was positive for COVID-19

Centrilobular nodules are present in those diseases involving centrilobular bronchiole, arteriole, or lymphatic. There is sparing of subpleural interestitium, with similar spaces between adjacent nodules [6]. In COVID-19 pneumonia, imaging findings of acute bronchiolitis with centrilobular nodules have been demonstrated [1] (Fig. 19).

**Fig. 20:**
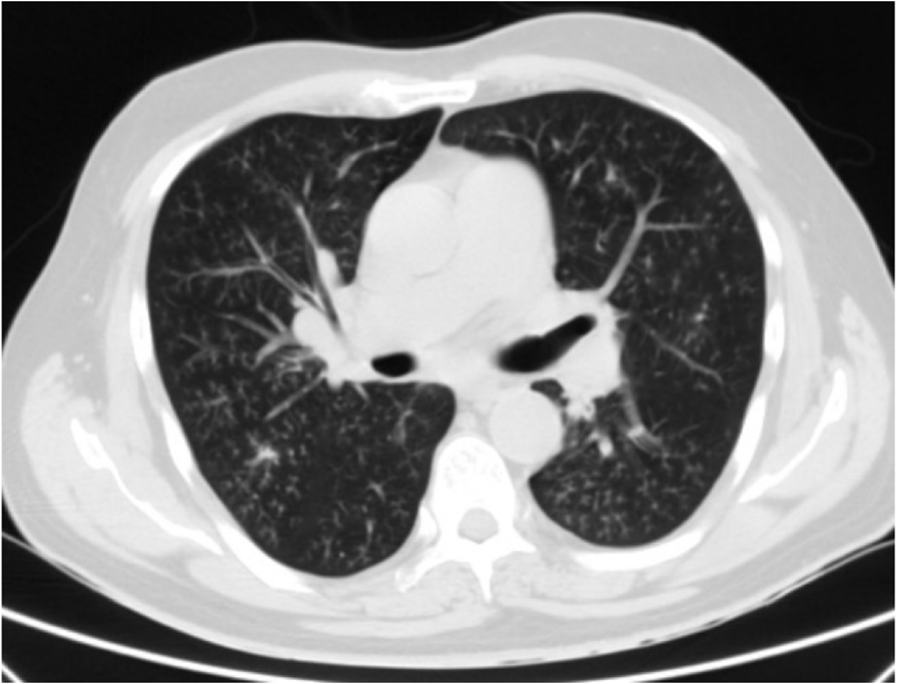
Infectious bronchiolitis: thin section CT shows diffuse clustered branching tree-in-bud opacities

**Fig. 21:**
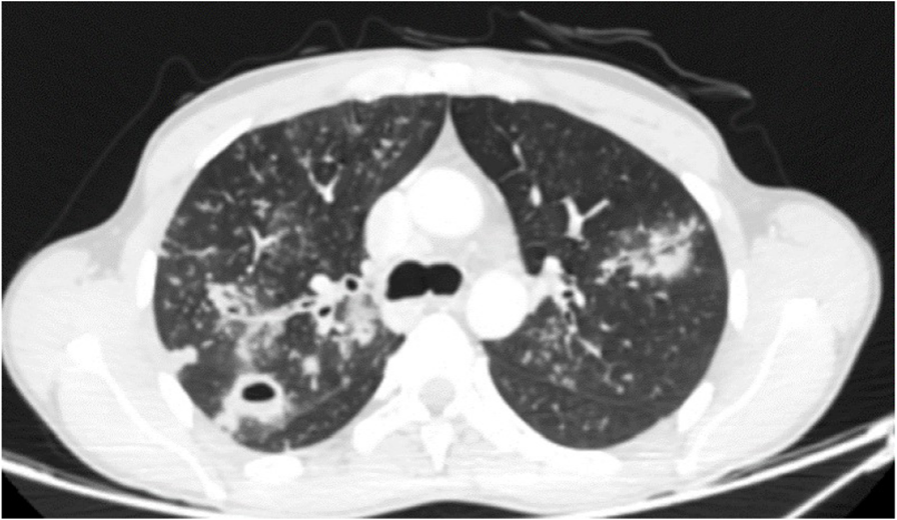
Post primary pattern of tuberculosis: thin section CT shows bilateral tree-in-bud opacities and a cavitary masslike consolidation in the right upper lobe

Differential diagnosis is broad, which includes different etiologies. Although bronchiolitis is the most common cause of centrilobular nodules [6], the most common type of bronchiolitis is infectious bronchiolitis, which can be classified as acute or chronic. In addition, acute bronchiolitis is typically viral or bacterial (staphylococcus) (Fig. 20), and chronic bronchiolitis is frequently mycobacterial (Fig. 21). Acute infectious bronchiolitis in CT scan often manifests itself as centrilobular nodules with a tree in bud appearance and peribronchial thickening [25].

## Conclusion

The imaging findings in this viral pneumonia showed a broad spectrum, which indicate a considerable overlap with various infectious and non-infectious etiologies. So, there are no pathognomonic imaging findings for COVID-19 pneumonia. Although CT scan is not a diagnostic and screening tool, familiarity with different imaging findings and their differential diagnosis can be helpful in rapid and accurate decision-making.

## Data Availability

Not applicable.

## Abbreviations

COVID-19: Novel corona virus2019
RT-PCR: Real-time polymerase chain reaction
CT: Computed tomography
CO-RADS: COVID-19 reporting and data system
GGO: Ground glass opacity
PAP: Pulmonary alveolar proteinosis
PTE: Pulmonary thromboembolism
OP: Organizing pneumonia
GVHD: Graft versus host disease
HRCT: High-resolution computed tomography
